# Changes in the Pneumococcal Vaccination Uptake and Its Determinants before, during, and after the COVID-19 Pandemic among Community-Living Older Adults in Hong Kong, China: Repeated Random Telephone Surveys

**DOI:** 10.3390/vaccines12080894

**Published:** 2024-08-07

**Authors:** Paul Shing-fong Chan, Josiah Poon, Soyeon Caren Han, Danhua Ye, Fuk-yuen Yu, Yuan Fang, Martin C. S. Wong, Phoenix K. H. Mo, Zixin Wang

**Affiliations:** 1 Jockey Club School of Public Health and Primary Care, Faculty of Medicine, The Chinese University of Hong Kong, Hong Kong, China; pchan@link.cuhk.edu.hk (P.S.-f.C.); danhuaye@cuhk.edu.hk (D.Y.); benfyyu@cuhk.edu.hk (F.-y.Y.); wong_martin@cuhk.edu.hk (M.C.S.W.); phoenix.mo@cuhk.edu.hk (P.K.H.M.); 2 School of Computer Science, The University of Sydney, Camperdown 2050, Australia; josiah.poon@sydney.edu.au; 3 School of Computing and Information Systems, The University of Melbourne, Parkville 3052, Australia; caren.han@unimelb.edu.au; 4 Department of Health and Physical Education, The Education University of Hong Kong, Hong Kong, China; lunajoef@gmail.com

**Keywords:** pneumococcal vaccination, COVID-19, community-living older adults, health belief model, random telephone survey

## Abstract

Pneumococcal vaccination (PV) is effective in preventing vaccine-type pneumococcal diseases. This study investigated the changes in PV uptake and its determinants before, during, and after the Coronavirus Disease 2019 (COVID-19) pandemic among community-living older adults aged ≥65 years in Hong Kong, China. Three rounds of random telephone surveys were conducted every two years from May 2019 to October 2023. Multivariate logistic regression models were fitted to examine the between-round differences in PV uptake rate and factors associated with PV uptake in each round. This study included 1563 participants. The standardized PV uptake rate in Round 1, 2, and 3 was 17.3%, 28.3%, and 35.5%, respectively. A significant difference in the PV uptake rate was found between Rounds 2 and 1 (*p* = 0.02), but not between Rounds 3 and 2 (*p* = 0.98). Perceived barriers, cue to action and self-efficacy, were significant determinants of PV uptake in all rounds. Perceived benefits were significant determinants of PV uptake in the first and second rounds, but not in the third round. Continuous monitoring of PV uptake and its determinants, and evaluating and adjusting the PV program, might contribute to the success of such a vaccination program in the post-pandemic era.

## 1. Introduction

The pneumococcal vaccination (PV) is effective in preventing vaccine-type non-invasive and invasive pneumococcal diseases among individuals aged ≥ 65 years without safety concerns [1,2,3,4]. In Hong Kong, older adults are recommended to take up the 15-valent pneumococcal conjugate vaccine (PCV15) or 23-valent pneumococcal polysaccharide vaccine (23vPPV) [5,6]. Older adults aged 65 years or above in Hong Kong could receive either free PV at public hospitals/clinics or subsidized PV at enrolled private clinics starting from October 2017 [5,6].

The most common co-infecting pathogen during the Coronavirus Disease 2019 (COVID-19) pandemic was Streptococcus pneumoniae [7]. The results of a meta-analysis indicated that PV was associated with a reduced risk of infection with SARS-CoV-2 [8]. In addition to reducing the burden on healthcare systems during the COVID-19 pandemic, vaccinating older adults against invasive pneumococcal diseases may also prevent some COVID-19 mortality from other co-infecting pathogens [8]. However, the PV coverage among all individuals aged 65 years or above in Hong Kong remained suboptimal (e.g., 33.9% in 2016 and 41.7% in 2023) [9], especially among community-living older adults (17.3% in 2019) [10].

The COVID-19 pandemic has had an impact on uptake and attitudes toward vaccination other than COVID-19 vaccines in different populations [11,12]. Numerous studies have investigated PV uptake among community-living older adults [13]. However, the majority of these studies were performed before the COVID-19 pandemic. We only identified two studies conducted during the COVID-19 pandemic. One survey showed that 91.5% of older adults in mainland China were willing to receive a PV [14]. In the United Kingdom, the coverage of PV was 67.7% among older adults [15]. We only found one Turkish study that reported an increase in PV uptake among older adults living in nursing homes after the COVID-19 outbreak (32.3% versus 5.5%) [16]. There was a dearth of studies investigating uptake, acceptance, or attitudes related to PV among community-living older adults in the post-pandemic era, or looking at the changes in these outcomes before, during, and after the COVID-19 pandemic.

To address the aforementioned knowledge gaps, we conducted three rounds of random telephone surveys to investigate the changes in the uptake and attitudes related to PV among community-living older adults before, during, and after the COVID-19 pandemic in Hong Kong, China. At each round of the survey, we also investigated determinants of PV uptake. The main hypotheses in this study were that the (1) PV uptake rate would increase over time, and that the (2) determinants of PV uptake would be different in different rounds of the survey.

## 2. Materials and Methods

### 2.1. Study Design

**Figure 1:**
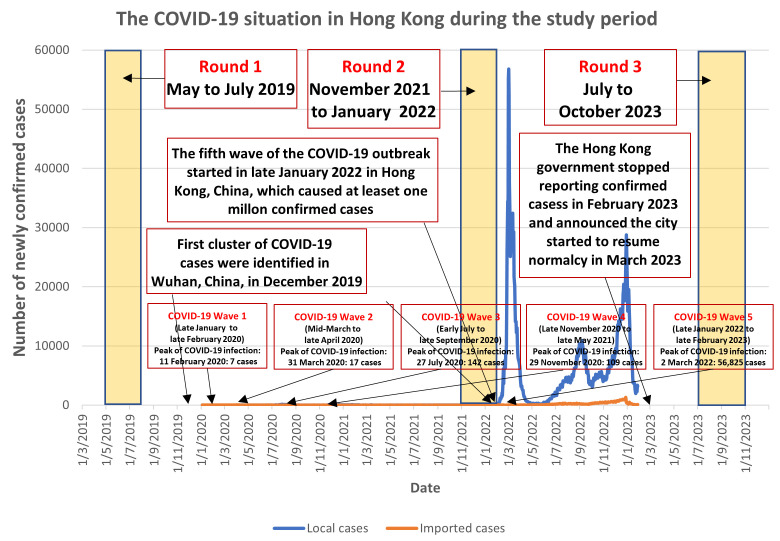
Context of this study.

This is an analysis of three rounds of random telephone surveys among community-living older adults aged 65 years or above in Hong Kong, China. The context of this study is shown in Figure 1. The first round was conducted before the COVID-19 outbreak from May to July 2019. There were four waves of COVID-19 outbreak between December 2019 and December 2021, and the peaks of these waves of outbreak were between 7 and 142 cases per day. The second round took place between November 2021 and January 2022. Hong Kong entered the fifth wave of COVID-19 outbreak soon after the completion of the second round (January 2022). The number of newly confirmed COVID-19 cases increased sharply and reached 30,000–56,825 cases per day at the peak of the fifth wave of outbreak. The third round of the survey was conducted from July to October 2023, after Hong Kong relieved all COVID-19 control measures and resumed normality in early 2023 [17].

### 2.2. Participants and Data Collection

Participants were (1) aged 65 years or above, (2) living in the community, (3) able to communicate in Cantonese, and (4) holders of a Hong Kong identity card. Older adults were excluded if they could not have effective communication with the interviewers. The same data collection method was used in each round of the survey [10]. First, we collected household telephone numbers listed in the most up-to-date telephone directories (about 350,000 numbers in total), and these telephone numbers were input into an Excel sheet. We then randomly selected numbers from the Excel sheet using simple random sampling (5500 numbers in the first round, and 4000 numbers in the second and third rounds). Participants were interviewed by trained interviewers from 6:00 p.m. to 10:00 p.m. on weekdays and from 2:00 p.m. to 9:00 p.m. on Saturdays. If no one answered the call after five attempts at different timeslots, such household would be considered as not having an eligible participant. Additionally, the one with a birthday closest to the survey date was invited to join the study when there were multiple individuals aged 65 years or above in the same household. Participants were briefed about the study and verbal consent was obtained before the interview. The Survey and Behavioral Research Ethics Committee of the Chinese University of Hong Kong approved this study (SBRE-19-183, SBRE-19-187, and SBRE-20-670).

### 2.3. Measures

#### 2.3.1. Questionnaire Development

The questionnaire was designed by a group of researchers from public health, behavioral health, and vaccination behaviors. We purposively recruited ten elderly people aged 65 years or above to test the clarity and readability of the questionnaire. The length of the questionnaire was considered acceptable, and the questions were easy to understand. Based on the minor comments suggested by the elderly, the questionnaire was refined.

#### 2.3.2. Background Characteristics

Participants reported information on socioeconomic characteristics, smoking and binge drinking behaviors in the past year, history of COVID-19 infection, history of seasonal influenza vaccination, number of doses of COVID-19 vaccination, and presence of high-risk conditions for severe invasive pneumococcal diseases.

#### 2.3.3. Uptake of and Attitudes toward PV

The uptake of any PV was used as the primary outcome. To measure attitudes toward PV, validated scales/items based on the health belief model (HBM) were used. The scales included the Perceived Benefit for Oneself Scale (3 items), Perceived Benefit for Others Scale (2 items), and Perceived Barrier Scale (3 items). The Cronbach’s alpha for these scales ranged from 0.77 to 0.90 in this study. In addition, cue to action and perceived self-efficacy were measured by two individual items. The response categories for the abovementioned items were 1 = disagree, 2 = neutral, and 3 = disagree.

### 2.4. Sample Size Planning

For Round 1, it was assumed that 10–15% of older adults in the reference group (without a facilitating condition of PV uptake) would receive PV, whereas 20% of all participants took up PV. We set a sample size of 750 for Round 1, and this would identify the smallest odds ratio of 1.61 with a power of 0.80 and an alpha value of 0.05 between older adults with and without a facilitating condition. We reduced the sample size to 400 in Rounds 2 and 3 due to the constraint of funding. Assuming that 20–40% of older adults received PV at each round, the current sample size could detect the smallest difference in PV uptake of 7.3% between Round 1 and Round 2 or 3, and 8.5% between Round 2 and Round 3. The sample size calculation was performed using PASS 11.0 (NCSS, LLC, Kaysville, UT, USA).

### 2.5. Statistical Analysis

Between-round pairwise comparison in terms of background characteristics, PV uptake, and attitudes toward PV was performed using the Chi-square tests or independent-sample *t*-tests. At each round of the survey, the PV uptake rate was standardized according to the age distribution of Hong Kong census data using the direct standardization method [18,19]. Pair-wise between-round comparisons in the uptake and attitudes related to PV were performed using logistic regression models (for uptake) or linear regression models (for attitudes), after adjusting for all background characteristics with significant between-round differences. Adjusted odds ratios (AORs), adjusted regression coefficient (adjusted β), and their 95% confidence interval (CI) were reported. In each round, univariate logistic regression models were used to measure the associations between the uptake of any PV and background characteristics. After that, taking significant background into account, a single logistic regression model was performed for one independent variable of interest (i.e., attitudes toward PV). To report the results, crude odds ratio (ORs), AORs, and 95% CIs were used and *p* < 0.05 was considered statistically significant. SPSS (version 26.0; IBM, Armonk, NY, USA) was used for data analysis.

## 3. Results

### 3.1. Background Characteristics

We called 5430 households in the first round, 3963 households in the second round, and 3670 households in the third round. The number of eligible households in each round was 1183 (Round 1), 683 (Round 2), and 574 (Round 3), respectively. Among these eligible households, 750 (Round 1), 440 (Round 2), and 373 (Round 3) completed the telephone interview. In the three rounds of the survey, the majority of the participants were female (56.8–61.1%) and married or cohabited with a partner (70.8–74.0%). Compared to other rounds, participants in the first round were older, less likely to receive tertiary education, more likely to be unemployed/retired, and refuse to disclose their income level. About 60% of the participants in each round reported having at least one high-risk condition of invasive pneumococcal diseases (Table 1).

### 3.2. Between-Round Difference in PV Uptake

The PV uptake rate was 17.3%, 25.2%, and 27.1% in the first, second, and third rounds of the survey, respectively, whilst the standardized uptake rates were 17.3% (Round 1), 28.3% (Round 2), and 35.5% (Round 3) (Supplementary Materials S1). After adjusting background variables with between-round difference, participants in the second and third rounds reported higher uptake comparing those in the first round (Round 2 vs. Round 1: AORs: 1.52, 95% CI: 1.06, 2.18, *p* = 0.02; Round 3 vs. Round 1: AORs: 1.92, 95% CI: 1.30, 2.83, *p* < 0.001). However, there was no significant increase in PV uptake when comparing the figures reported in the third versus the second round (AORs: 0.99, 95% CI: 0.42, 2.35, *p* = 0.98) (Table 2).

### 3.3. Between-Round Difference in Attitudes toward PV

Compared to participants in the first round, those in the second and third rounds perceived more benefits of PV for themselves and for others. Participants in the third round perceived more benefits of PV for themselves but not for others compared to those in the second round. Compared to participants in the first round, those in the second round received lower cue to action related to PV uptake. The item score of the cue to action in the third round was similar to that of the first round but was significantly higher than that observed in the second round. There was no significant between-round difference in perceived barrier or perceived self-efficacy to receive PV (Table 2).

### 3.4. Factors Associated with PV Uptake

History of seasonal influenza vaccination was associated with higher PV uptake in all rounds of the surveys. Older age, with full-time/part-time employment, and having self-reported at least one high-risk condition of invasive pneumococcal diseases were positively correlated with PV uptake in both the second and third rounds of the survey, but not in the first round. Sex assigned at birth, current relationship status, monthly household income, living alone, receiving CSSA, and history of COVID-19 were associated with PV uptake in the third round but not in other rounds (Table 3).

After adjusting significant background characteristics, perceiving more barriers was associated with lower PV uptake (AORs: 0.25 to 0.33, 95%CI: 0.19, 0.34 to 0.26, 0.42, all *p* < 0.001), while receiving more suggestions from significant others (cue to action) (AORs: 3.69 to 16.17; 95%CI: 2.28, 5.98 to 7.25, 32.91, all *p* < 0.001) and having higher self-efficacy (AORs: 6.05 to 8.13; 95%CI: 3.12, 11.74 to 3.75, 17.63, all *p* < 0.001) were associated with higher uptake in all rounds of the surveys. Perceiving more benefits of PV for oneself (Round 1: AORs: 3.31, 95%CI: 2.48, 4.41, *p* < 0.001; Round 2: AORs: 2.54, 95%CI: 1.97, 3.27, *p* < 0.001) and for others (Round 1: AORs: 2.64, 95%CI: 2.09, 3.34, *p* < 0.001; Round 2: AORs: 2.32, 95%CI: 1.73, 3.11, *p* < 0.001) were associated with higher PV uptake in both the first and the second rounds of the survey. However, the associations between these two variables and PV uptake were statistically non-significant in the third round of the survey (perceived benefit for oneself: AORs: 1.37, 95%CI: 0.98, 1.50, *p* = 0.06; perceived benefit for others: AORs: 1.22, 95%CI: 0.86, 1.72, *p* = 0.26) (Table 4).

## 4. Discussion

This is one of the first studies investigating changes in uptake, attitudes, and determinants related to PV among older adults before, during, and after the COVID-19 pandemic. This study addressed the potential knowledge gaps with the strengths of having random and population-based samples and using the HBM to guide the variable selection. Our findings might provide evidence of the impact of COVID-19 and governmental programs on PV uptake among community-living older adults in Hong Kong and a knowledge basis to inform service planning.

The PV uptake rate in all rounds of the survey was much lower than some developed countries (e.g., Australia, the United States, and Canada) [20,21,22]. The PV uptake rate during the pandemic was significantly higher than the time before COVID-19. During the COVID-19 pandemic, information emphasizing the importance of vaccination was widely spread on different media channels [23,24], which might increase older adults’ awareness and motivation to use vaccines to prevent infectious diseases. However, the increase in PV uptake when comparing the post-pandemic era with the time during the pandemic was relatively small (1.9%) and statistically non-significant. It is possible that older adults no longer perceive infectious diseases as serious health threats in the post-pandemic era. In addition, the need for COVID-19 vaccine booster doses and the rapidly changing vaccine guidelines during the pandemic might increase people’s inaction toward vaccination instructions due to perceived burden and burnout, a phenomenon known as vaccine fatigue [25].

Similar to previous studies, a history of seasonal influenza vaccination was associated with PV in all rounds of the survey [26]. In Hong Kong, it is a common practice for healthcare providers to suggest older adults receive PV when they are taking up seasonal influenza vaccination [27]. In line with our hypothesis, factors associated with PV uptake were not the same at different time points. Having full-time/part-time employment and high-risk conditions of invasive pneumococcal diseases were associated with higher PV during and after the COVID-19 pandemic, but not in the time before COVID-19. Based on the lessons learned after the COVID-19 outbreak, older adults with employment might realize that they had a high risk of infectious diseases at work due to frequent interpersonal interactions. Through years of health promotion efforts, it is possible that more elderly understand that PV is especially important for people with high-risk conditions. In addition to these factors, programs promoting PV in the post-pandemic era should pay more attention to older adults who are male, married, or cohabite with a partner, and with history of COVID-19 infection, as they are less likely to receive such vaccination.

Our findings also provided some empirical implications for developing interventions promoting PV in the post-pandemic era. Compared to the time before COVID-19, both the perceived benefits of PV for oneself and for others increased significantly during the COVID-19 pandemic. Health promotion efforts made by the government might have caused some of these changes. Similar to other studies, perceived benefits for oneself and for others were associated with higher PV uptake in both the first and second rounds of the survey [28]. Therefore, changes in perceived benefit might have contributed to the increase in PV uptake comparing the second with the first round of the survey. Increasing perceived benefits might not be a useful health promotion strategy in the post-pandemic era, as perceived benefits were not significant determinants of PV uptake in the third round.

Compared to the time before COVID-19, older adults received fewer cues to action to receive PV during the pandemic. The level of cue to action increased significantly and was similar to the time before COVID-19 in the post-pandemic era. Previous studies suggested that healthcare professionals, family members, and friends are significant others of older adults regarding vaccination uptake [29,30]. During the COVID-19 pandemic, PV for older adults might not be the top priority of health service providers [31]. Meanwhile, the COVID-19 situation and vaccination might have attracted the attention of most family members and friends of older adults. When Hong Kong lifted COVID-19 control measures, health service providers could resume their routine work (including PV promotion). Future programs should keep involving these significant others of older adults to provide a strong cue to action, as such a construct was associated with higher PV in all three time points.

The COVID-19 pandemic might have little impact on perceived barriers and perceived self-efficacy related to PV uptake, as the level of both constructs was similar across time points. It is necessary to reduce perceived barriers and to increase perceived self-efficacy as both constructs were significant determinants of PV uptake at all three time points. Currently, free PV is provided to older adults with high-risk conditions and low socioeconomic status. Other older adults can only receive subsidized vaccines at private clinics. The government should also consider expanding the eligibility for free PV to further reduce the barriers related to cost. In order to reduce the concerns related to side effects, positive experiences shared by vaccinated older adults might be useful, as it is common for older adults to believe that the experiences and information of their peers are trustworthy [32]. Furthermore, to increase the perceived self-efficacy of older adults, it might be beneficial to facilitate the development of a concrete action plan to receive PV [33].

This study had several limitations. First, those aged ≥75 years were under-sampled in the second and third rounds of the survey. Since older age was associated with higher PV uptake, the uptake rate in the second and third rounds of the surveys might be underestimated. Second, due to the constraint of funding, the sample size of the second and third rounds of the survey was smaller than the first round of the survey. The reduction in sample size reduced the power to identify the difference in PV uptake between the second and third rounds. However, the effect size (AORs) representing the difference in PV uptake between the second and third rounds was quite small. Therefore, the non-significance cannot be fully explained by the relatively small sample size. Third, we did not include variables measuring the direct impact of COVID-19 or COVID-19 vaccination on PV uptake. The interim recommendation in Hong Kong on the use of COVID-19 vaccines released in February 2021 stated that the administration of COVID-19 vaccination should be 14 days before or after another prophylactic vaccine (including PV) to allow for the clearer ascertainment of potential adverse effects [34]. The recommendation was updated in August 2022, and co-administration of PV and COVID-19 vaccination is now recommended [35]. It was likely that participants in the second round had concerns about the potential interaction between COVID-19 vaccination and PV, which might be a barrier for them to receive PV. Fourth, all data were self-reported, and verification was difficult. Recall bias might exist. Fifth, our response rate was 60% in each round of the survey, which was comparable to previous random telephone surveys on similar topics in Hong Kong [36,37]. However, we were not able to collect the information of older adults who refused to join the study. Hence, selection bias existed. Furthermore, the cross-sectional study design could not establish a causal relationship between the independent and dependent variables.

## 5. Conclusions

The increase in the PV uptake rate was small and non-significant when comparing the post-pandemic era with the time during the COVID-19 pandemic. Effective interventions promoting PV are needed. Determinants of PV uptake were not the same in the post-pandemic era compared to the time during or before COVID-19. Health promotion of PV in the post-pandemic era should consider reducing barriers related to cost, side effects, and inconvenience, providing cue to action and increasing perceived self-efficacy. Continuously monitoring PV uptake and its determinants, and evaluating and adjusting the PV program, might contribute to the success of such vaccination programs.

## Figures and Tables

**Table 1:** Background characteristics of community-dwelling older adults in Hong Kong in the three waves of random telephone surveys.

	Round 1 (n = 750)	Round 2 (n = 440)	Round 3 (n = 373)	Round 2 vs. Round 1	Round 3 vs. Round 1	Round 3 vs. Round 2
	n (%)	n (%)	n (%)	*p* Values	*p* Values	*p* Values
Sociodemographic characteristics						
Age (years), mean (SD)	74.8 (8.0)	70.4 (4.5)	69.6 (4.4)	<0.001	<0.001	0.01
Sex assigned at birth						
Male	295 (39.3)	171 (38.9)	161 (43.2)	0.87	0.22	0.21
Female	455 (60.7)	269 (61.1)	212 (56.8)			
Highest education level attained						
Secondary or below	725 (96.7)	396 (90.0)	307 (82.3)	<0.001	<0.001	<0.001
Tertiary or above	25 (3.3)	44 (10.0)	66 (17.7)			
Married or cohabited with a partner						
No	219 (29.2)	113 (25.7)	108 (29.0)	0.19	0.93	0.30
Yes	531 (70.8)	327 (74.3)	265 (71.0)			
Having full-time/part-time employment						
Yes	38 (5.1)	63 (14.3)	62 (16.6)	<0.001	<0.001	0.36
No	712 (94.9)	377 (85.7)	311 (83.4)			
Average household income per month, HKD (USD)						
<20,000 (2564)	423 (56.4)	311 (70.7)	170 (45.6)	<0.001	<0.001	<0.001
≥20,000 (2564)	25 (3.3)	58 (13.2)	95 (25.5)			
No stable income	65 (8.7)	17 (3.9)	38 (10.2)			
Refuse to disclose	237 (31.6)	54 (12.3)	70 (18.8)			
Living with other household members						
Yes	613 (81.7)	359 (81.6)	314 (84.2)	0.95	0.31	0.33
No	137 (18.3)	81 (18.4)	59 (15.8)			
Receiving Comprehensive Social Security Assistance (a governmental financial support scheme providing a safety net for Hong Kong residents who cannot support themselves financially)						
No	712 (94.9)	408 (92.7)	355 (95.2)	0.12	0.86	0.15
Yes	38 (5.1)	32 (7.3)	18 (4.8)			
Self-reported high-risk conditions for invasive pneumococcal diseases, Yes						
Invasive pneumococcal diseases	2 (0.3)	7 (1.6)	4 (1.1)			
Cerebrospinal fluid leakage	2 (0.3)	0 (0)	0 (0)			
Hypertension	445 (59.3)	212 (48.2)	178 (47.7)			
Chronic cardiovascular diseases	65 (8.7)	46 (10.5)	44 (11.8)			
Chronic lung diseases	1 (0.1)	8 (1.8)	4 (1.1)			
Chronic liver diseases	0 (0)	10 (2.3)	8 (2.1)			
Chronic kidney diseases	5 (0.7)	3 (0.7)	2 (0.5)			
Diabetes mellitus	139 (18.5)	83 (18.9)	73 (19.6)			
Immunocompromised states	1 (0.1)	5 (1.1)	5 (1.3)			
Any of above	483 (64.4)	271 (61.6)	222 (59.5)	0.33	0.11	0.55
Had ever been infected with COVID-19 in their lifetime						
No	NA	432 (98.2)	148 (39.7)	NA	NA	<0.001
Yes	NA	8 (1.8)	225 (60.3)			
History of other vaccination						
Had ever received seasonal influenza vaccination in lifetime						
No	372 (49.6)	176 (40.0)	137 (36.7)	<0.001	<0.001	0.34
Yes	378 (50.4)	264 (60.0)	236 (63.3)			
Number of doses of COVID-19 vaccination received by the participants						
0 dose	NA	173 (39.3)	15 (4.0)	NA	NA	<0.001
1 dose	NA	10 (2.3)	3 (0.8)			
2 doses	NA	257 (58.4)	31 (8.3)			
≥3 doses	NA	0 (0)	324 (86.9)			

NA: not applicable.

**Table 2:** Comparing PV uptake and attitudes toward PV between participants in Rounds 1, 2, and 3.

	Round 1 (n = 750)	Round 2 (n = 440)	Round 3 (n = 373)	Round 2 vs. Round 1	Round 3 vs. Round 1	Round 3 vs. Round 2
	n (%)	n (%)	n (%)	AORs ^1^ (95%CI; *p* Values)	AORs ^2^ (95%CI; *p* Values)	AORs ^3^ (95%CI; *p* Values)
PV uptake						
Uptake of at least one dose of PV, Yes	130 (17.3)	111 (25.2)	101 (27.1)	1.52 (1.06, 2.18; * p * = 0.02)	1.92 (1.30, 2.83; * p * < 0.001)	0.99 (0.42, 2.35, *p* = 0.98)
				Adjusted β ^1^ (95%CI; *p* values)	Adjusted β ^2^ (95%CI; *p* values)	Adjusted β ^3^ (95%CI; *p* values)
Attitudes toward PV						
Perceived benefit of PV for oneself, agree						
PV is highly effective in preventing you from pneumonia	326 (43.5)	206 (46.8)	297 (79.6)			
PV is highly effective in preventing you from severe invasive pneumococcal diseases (e.g., septicemia or meningitis)	107 (14.3)	162 (36.8)	279 (74.8)			
You will feel at ease after taking up PV	393 (52.4)	227 (51.6)	284 (76.1)			
Perceived Benefit for Oneself Scale ^#^, mean (SD)	6.77 (1.50)	7.20 (1.51)	8.16 (1.28)	0.33 (0.14, 0.52; * p * < 0.001)	1.21 (1.01, 1.40; * p * < 0.001)	0.52 (0.21, 0.83; * p * = 0.001)
Perceived benefits of PV for others, agree						
Taking up PV is highly effective in preventing pneumonia transmission in Hong Kong	258 (34.4)	202 (45.9)	264 (70.8)			
Taking up PV is highly effective in protecting your family members against pneumonia	263 (35.1)	198 (45.0)	240 (64.3)			
Perceived Benefit for Others Scale ^#^, mean (SD)	4.58 (1.11)	4.85 (1.04)	5.22 (1.08)	0.16 (0.03, 0.30; * p * = 0.02)	0.46 (0.31, 0.61; * p * < 0.001)	0.14 (−0.09, 0.38; *p* = 0.23)
Perceived barriers of taking up PV, agree						
PV is expensive for you	121 (16.1)	41 (9.3)	74 (19.8)			
You concerned about side effects of PV	142 (18.9)	27 (6.1)	56 (15.0)			
The time and venue of PV is inconvenient for you	63 (8.4)	15 (3.4)	26 (7.0)			
Perceived Barrier Scale ^#^, mean (SD)	5.11 (1.64)	4.93 (1.24)	4.83 (1.36)	−0.01 (−0.20, 0.18; *p* = 0.92)	0.02 (−0.18, 0.23; *p* = 0.82)	−0.007 (−0.30, 0.28; *p* = 0.96)
Your significant others suggested you take up PV (cue to action), agree	330 (44.0)	117 (26.6)	202 (54.2)			
Item score ^#^, mean (SD)	2.31 (0.68)	2.18 (0.57)	2.46 (0.65)	−0.21 (−0.29, −0.13; * p * < 0.001)	0.05 (−0.04, 0.14; *p* = 0.26)	0.16 (0.02, 0.29; * p * = 0.02)
You are confident to take up PV if you want to (self-efficacy), agree	457 (60.9)	275 (62.5)	290 (77.7)			
Item score ^#^, mean (SD)	2.42 (0.79)	2.58 (0.57)	2.69 (0.62)	0.03 (−0.06, 0.12; *p* = 0.53)	0.09 (−0.01, 0.18; *p* = 0.09)	0.05 (−0.08–0.18, *p* = 0.46)

AORs: adjusted odds ratio. Adjusted β: adjusted regression coefficient. CI: confidence interval. ^1^ Odds ratios or regression coefficient adjusted for age, highest education level attained, current employment status, monthly household income, history of seasonal influenza vaccination. ^2^ Odds ratios or regression coefficient adjusted for age, highest education level attained, current employment status, monthly household income, history of seasonal influenza vaccination. ^3^ Odds ratios or regression coefficient adjusted for age, highest education level attained, monthly household income, history of COVID-19 infection, number of doses of COVID-19 vaccination received.

**Table 3:** Associations between background characteristics and PV uptake in each round of survey.

	Round 1		Round 2		Round 3	
	ORs (95%CI)	*p* Values	ORs (95%CI)	*p* Values	ORs (95%CI)	*p* Values
Age (years)	1.02 (0.99, 1.05)	0.07	1.08 (1.03, 1.13)	0.002	1.23 (1.16, 1.30)	<0.001
Sex assigned at birth						
Male	1.00		1.00		1.00	
Female	0.86 (0.59, 1.26)	0.45	1.53 (0.97, 2.42)	0.07	1.95 (1.20, 3.15)	0.01
Highest education level attained						
Secondary or below	1.00		1.00		1.00	
Tertiary or above	0.91 (0.31, 2.68)	0.86	1.81 (0.94, 3.49)	0.08	0.84 (0.45, 1.55)	0.57
Married or cohabited with a partner						
No	1.00		1.00		1.00	
Yes	1.39 (0.90, 2.15)	0.14	0.86 (0.53, 1.39)	0.53	0.48 (0.30, 0.78)	0.003
Having full-time/part-time employment						
Yes	1.00		1.00		1.00	
No	0.40 (0.12, 1.30)	0.13	0.27 (0.11, 0.65)	0.003	0.24 (0.10, 0.59)	0.002
Average household income per month, HKD (USD)						
<20,000 (2564)	1.00		1.00		1.00	
≥20,000 (2564)	0.82 (0.27, 2.45)	0.72	0.94 (0.48, 1.84)	0.86	0.35 (0.18, 0.68)	0.002
No stable income	0.97 (0.50, 1.90)	0.93	2.28 (0.84, 6.21)	0.11	2.52 (1.23, 5.14)	0.01
Refuse to disclose	0.72 (0.46, 1.11)	0.14	1.63 (0.87, 3.04)	0.13	0.34 (0.16, 0.71)	0.004
Living with other household members						
Yes	1.00		1.00		1.00	
No	0.53 (0.30, 1.02)	0.06	1.32 (0.77, 2.25)	0.31	2.98 (1.68, 5.30)	<0.001
Receiving Comprehensive Social Security Assistance ^a^						
No	1.00		1.00		1.00	
Yes	0.55 (0.19, 1.57)	0.26	0.67 (0.27, 1.66)	0.38	4.63 (1.74, 12.30)	0.002
Self-reported having any high-risk conditions for invasive pneumococcal diseases						
No	1.00		1.00		1.00	
Yes	1.10 (0.74, 1.64)	0.65	1.98 (1.23, 3.17)	0.01	1.78 (1.10, 2.90)	0.02
Had ever been infected with COVID-19 in their lifetime						
No			1.00		1.00	
Yes	N.A.	N.A.	0.99 (0.20, 4.97)	0.99	0.57 (0.36, 0.91)	0.02
Had ever received seasonal influenza vaccination in lifetime						
No	1.00		1.00		1.00	
Yes	46.00 (16.78, 126.08)	<0.001	39.92 (12.42, 128.30)	<0.001	48.78 (11.79, 201.77)	<0.001
Number of doses of COVID-19 vaccination received by the participants						
0 dose			1.00		1.00	
1 dose			1.15 (0.23, 5.66)	0.87	N.A.	N.A.
2 doses			2.00 (1.25, 3.20)	0.004	0.96 (0.20, 4.51)	0.96
≥3 doses	N.A.	N.A.	N.A.	N.A.	1.59 (0.44, 5.75)	0.48

a: comprehensive Social Security Assistance is a governmental financial support scheme providing a safety net for Hong Kong residents who cannot support themselves financially. CI: confidence interval; N.A.: not applicable; ORs: crude odds ratio.

**Table 4:** Factors associated with PV uptake in each round of survey.

	Round 1		Round 2		Round 3	
	AORs ^1^ (95%CI)	*p* Values	AORs ^2^ (95%CI)	*p* Values	AORs ^3^ (95%CI)	*p* Values
Perceived Benefit for Oneself Scale	3.31 (2.48, 4.41)	<0.001	2.54 (1.97, 3.27)	<0.001	1.37 (0.98, 1.90)	0.06
Perceived Benefit for Others Scale	2.64 (2.09, 3.34)	<0.001	2.32 (1.73, 3.11)	<0.001	1.22 (0.86, 1.72)	0.26
Perceived Barrier Scale	0.33 (0.26, 0.42)	<0.001	0.25 (0.19, 0.34)	<0.001	0.31 (0.22, 0.42)	<0.001
Your significant others suggested you take up PV (cue to action)	16.17 (7.95, 32.91)	<0.001	3.69 (2.28, 5.98)	<0.001	3.93 (2.05, 7.56)	<0.001
You are confident to take up PV if you want to (self-efficacy)	6.05 (3.12, 11.74)	<0.001	8.13 (3.75, 17.63)	<0.001	6.84 (2.18, 21.43)	<0.001

AORs: adjusted odds ratio. Adjusted β: adjusted regression coefficient. CI: confidence interval. ^1^ Odds ratios adjusted for whether they had ever received seasonal influenza vaccination in lifetime. ^2^ Odds ratios adjusted for age, whether having full-time/part-time employment, self-reported having any high-risk conditions for invasive pneumococcal diseases, whether they had ever received seasonal influenza vaccination in lifetime, number of doses of COVID-19 vaccination received. ^3^ Odds ratios adjusted for age, sex assigned at birth, whether they married or cohabited with a partner, whether having full-time/part-time employment, average household income per month, whether living with other household members, whether receiving Comprehensive Social Security Assistance, self-reported having any high-risk conditions for invasive pneumococcal diseases, whether they had ever been infected with COVID-19 in lifetime, and whether they had ever received seasonal influenza vaccination in lifetime.

## Data Availability

The data presented in this study are available from the corresponding author upon request. The data are not publicly available as they contain personal behaviors.

## Supplementary Materials

The following supporting information can be downloaded at: https://www.mdpi.com/article/10.3390/vaccines12080894/s1, Supplementary Materials Material S1: Standardization of PV uptake rate among the elderly aged 65 years or above in Hong Kong.
