# Supplementary material for: Changes in the Pneumococcal Vaccination Uptake and Its Determinants before, during, and after the COVID-19 Pandemic among Community-Living Older Adults in Hong Kong, China: Repeated Random Telephone Surveys
Source: Vaccines (Basel). 2024 Aug 7;12(8):894. doi: 10.3390/vaccines12080894 (PMC11359888; doi:10.3390/vaccines12080894)
Supplement: Supplementary file 1 [file vaccines-12-00894-s001.zip › vaccines-3121706-supplementary.pdf]

Supplementary Material S1: Standardization of PV uptake rate among the elderly aged 65 years or above in Hong Kong

	Round 1				Round 2				Round 3			
Age (years)	(a) Number of the elderly in Hong Kong *	(b) PV uptake rate in the present study	(c) Standardized number of elderly taking up PV (a*b)	Standardized PV uptake rate [total of (c) / total of (a)]	(d) Number of the elderly in Hong Kong *	(e) PV uptake rate in the present study	(f) Standardized number of elderly taking up PV (d*e)	Standardized PV uptake rate [total of (f) / total of (d)]	(g) Number of the elderly in Hong Kong *	(h) PV uptake rate in the present study	(i) Standardized number of elderly taking up PV (g*h)	Standardized PV uptake rate [total of (i) / total of (g)]
65-69	444000	9.1%	40404		508300	17.4%	88444		568500	10.9%	61967	
70-74	307600	24.3%	74747		381400	32.0%	122048		424800	47.5%	201780	
75 or above	570400	19.9%	113510		604707	35.1%	212252		648600	49.2%	319111	
				17.3%				28.3%				35.5%

* The number of elderly in each age group in each round was collected from the Hong Kong Census and Statistics Department.

PV: pneumococcal vaccination
